# Genetic Variants in *BIRC5* (rs8073069, rs17878467, and rs9904341) Are Associated with Susceptibility in Mexican Patients with Breast Cancer: Clinical Associations and Their Analysis In Silico

**DOI:** 10.3390/genes16070786

**Published:** 2025-06-30

**Authors:** María Renee Jiménez-López, César de Jesús Tovar-Jácome, Alejandra Palacios-Ramírez, Martha Patricia Gallegos-Arreola, Teresa Giovanna María Aguilar-Macedo, Rubria Alicia González-Sánchez, Efraín Salas-González, José Elías García-Ortiz, Clara Ibet Juárez-Vázquez, Mónica Alejandra Rosales-Reynoso

**Affiliations:** 1 División de Medicina Molecular, Centro de Investigación Biomédica de Occidente (CIBO), Instituto Mexicano del Seguro Social (IMSS), Sierra Mojada 800, Col. Independencia, Guadalajara 44340, Mexico; mreneejimenezlo@gmail.com (M.R.J.-L.); cesartovjacome@gmail.com (C.d.J.T.-J.); giovanna.0187@gmail.com (T.G.M.A.-M.); rubria18@gmail.com (R.A.G.-S.); 2 Servicio de Ginecología Oncológica, Unidad Médica de Alta Especialidad, Hospital de Ginecología y Obstetricia, Centro Médico Nacional de Occidente (CMNO), Instituto Mexicano del Seguro Social (IMSS), Guadalajara 44329, Jalisco, Mexico; alepr823@gmail.com; 3 División de Genética, Centro de Investigación Biomédica de Occidente (CIBO), Instituto Mexicano del Seguro Social (IMSS), Sierra Mojada 800, Col. Independencia, Guadalajara 44340, Mexico; marthapatriciagallegos08@gmail.com (M.P.G.-A.); jose.elias.garcia@gmail.com (J.E.G.-O.); 4 Dirección Académica Aparatos y Sistemas I, Facultad de Medicina, Decanato Ciencias de la Salud, Universidad Autónoma de Guadalajara (UAG), Zapopan 45129, Mexico; 5 Servicio de Oncología Médica, Unidad Médica de Alta Especialidad, Hospital de Ginecología y Obstetricia, Centro Médico Nacional de Occidente (CMNO), Instituto Mexicano del Seguro Social (IMSS), Guadalajara 44329, Jalisco, Mexico; esgonco@hotmail.com

**Keywords:** breast cancer, *BIRC5*, genotypes, haplotypes, susceptibility

## Abstract

Background/Objectives: Breast cancer (BC) is a multifactorial disease, with genetic alterations in cell proliferation and migration pathways being significant risk factors. This study examines the association between three variants in the *BIRC5* gene (rs8073069, rs17878467, and rs9904341) and breast cancer (BC) susceptibility. Methods: Peripheral blood DNA samples were collected from 423 women (221 BC patients and 202 healthy controls). Genotyping was performed by polymerase chain reaction restriction fragment length polymorphism (PCR-RFLP) methodology. Associations were calculated using odds ratios (OR), with *p*-values adjusted by the Bonferroni test (significance at *p* ≤ 0.016). In silico analyses were conducted to predict the functional impact of the analyzed variants. Results: Patients carrying the C/C genotype for the rs8073069 variant showed increased susceptibility to BC with early TNM (tumor-node-metastasis classification) stage and Luminal A subtype (OR > 2.00; *p* ≤ 0.004). For the rs17878467 variant, patients with the C/T or T/T genotype exhibited a higher susceptibility to developing breast cancer (BC), particularly at early TNM stages or with a histological lobular type (OR > 2.00; *p* ≤ 0.012). Regarding the rs9904341 variant, patients with the G/C or C/C genotype had a higher susceptibility to breast cancer. Notably, G/C genotype carriers with Luminal A and B subtypes, and C/C genotype carriers who had TNM stages II and III, and Luminal A, Luminal B, and HER2 subtypes demonstrated increased risk (OR > 2.00; *p* ≤ 0.009). The C-T-C haplotype (rs8073069–rs17878467–rs9904341) was significantly associated with BC (OR = 4.20; 95% CI = 2.38–7.41; *p* ≤ 0.001). In silico analysis using CADD indicated a low probability of deleterious effects. Conclusions: The results suggest that the rs8073069, rs17878467, and rs9904341 variants in *BIRC5* have a significant influence on breast cancer susceptibility.

## 1. Introduction

Breast cancer (BC) is one of the most common malignancies among women worldwide. According to GLOBOCAN, in 2022, there were 2,296,840 new cases globally, which caused the death of approximately 666,103 women [1]. In Mexico, according to the Instituto Nacional de Estadística y Geografía (INEGI) [2], BC causes the majority of malignant tumor-related deaths in women aged 20 and older [3].

A complex combination of environmental and genetic factors in BC contributes to its prevalence. Some of the common risk factors include genetic background, age, menstrual history, hormonal and reproductive factors, excessive alcohol or tobacco consumption, radiation, benign breast disease, and obesity [4]. Alteration of apoptosis is a common phenomenon in cancer cells and is crucial in the development, progression, and metastasis [5].

Baculoviral inhibitor of apoptosis repeat-containing 5 (*BIRC5*), also known as “survivin”, a protein encoded by the *BIRC5* gene, is a member of the inhibitor of apoptosis (IAP) family [6]. Survivin is a small protein with various isoforms, most of which are related to inhibiting apoptosis and promoting cell proliferation [7]. Survivin is involved in several molecular networks linked to cancer processes, including tumor cell proliferation, invasive growth, and distant metastasis. Survivin is highly expressed in many cancers, including breast cancer (BC), and its expression levels correlate with tumor stage, prognosis, and response to therapy [8,9,10,11].

Bioinformatics studies using independent datasets have demonstrated that survivin is essential in breast cancer (BC) development [8,9]. However, the clinical significance and molecular mechanisms underlying survivin’s involvement in breast cancer (BC) development remain unclear.

The *BIRC5* gene is located on chromosome 17q25.3 and comprises four exons and five introns, encoding ten splice variants, seven of which have known functions [10]. *BIRC5* has a TATA box-less promoter with a canonical CpG island, one cell cycle homology region (CHR), and three cell-cycle dependent elements (CDE) characteristic of G2M-expressed genes. [11,12,13] CHR or CDE region deletion in *BIRC5* promoter leads to loss of cell-cycle dependent expression needed for the basal transcriptional activation of survivin expression [12,14].

Approximately 199 variants in the *BIRC5* gene have been identified, with the most frequently studied being those located in the promoter region of *BIRC5*, including the −625 G>C (rs8073069), −241 C>T (rs17878467), and −31 G>C (rs9904341) [15]. Alteration of gene expression has been implicated in these regulatory variants, which are located in promoter or 5′ UTR. Some variants create binding sites for transcription factors or regulatory miRNA, potentially influencing survivin levels [15].

Several studies have suggested that variants in the *BIRC5* gene may serve as potential genetic markers for cancer susceptibility as: endometrial [16], prostate [17,18], gastrointestinal [19], bladder [20,21], colorectal [22], ovarian cancer [12], oropharyngeal squamous cell [6], hepatocellular [23], nasopharyngeal [24], renal cell cancer [25], chronic myeloid leukemia [26], non-small cell lung [27], and breast cancer [5,28]. However, studies that include the rs8073069 (−625 G>C), rs17878467 (−241 C>T), and rs9904341 (−31 G>C) variants in *BIRC5* and BC risk have shown contradictory results among different populations [5,28,29,30,31]. For this reason, BC risk studies in the Mexican population regarding these variants in the *BIRC5* gene remain unknown. Therefore, this study aims to investigate, for the first time, the allele, genotype, and haplotype distribution and association of the −625 G>C (rs8073069), −241 C>T (rs17878467), and −31G>C (rs9904341) variants in the *BIRC5* gene with breast cancer in Mexican women, as well as their clinicopathological characteristics.

## 2. Materials and Methods

### 2.1. Subjects

A total of 423 women were recruited, 221 patients with clinical and histological diagnosis of breast cancer based on the criteria of the UMAE Hospital de Gineco-Obstetricia of the Instituto Mexicano del Seguro Social (IMSS) in Guadalajara, Jalisco, Mexico. Clinical stage classification for Breast cancer was realized according to the tumor-node-metastasis (TNM). For the control group, 202 healthy women, unrelated and age-matched to the patient group, were included. 

The BC group comprised women with any stage of BC, aged 18 years or older, and any treatment status or therapeutic stage. The control group consisted of healthy female donors from the general Mexican population aged 18 years or older. Women with a previous cancer history were excluded from the control group. The National Committee for Scientific Research of the Instituto Mexicano del Seguro Social (IMSS) approved the study (R-2020-785-130). It ensured that it was conducted by national and international ethical standards. All the participants signed informed consent for participation in this study. Information about the epidemiological, clinical, and pathological features of the patients and/or the control group was obtained from hospital records and an epidemiological questionnaire, completed before the sample was taken.

### 2.2. Genotyping

Genomic DNA from peripheral blood lymphocytes was isolated using the Miller, Dykes & Polesky method [32]. The variants rs8073069 (G>C), rs17878467 (C>T), and rs9904341 (G>C) in the *BIRC5* gene were genotyped by polymerase chain reaction-restriction fragment length polymorphism (PCR-RFLP) methodology. For rs8073069 variant was performed using the forward primer F: 5′-GTA CAT TTG TCC TTC ATG CGC-3′ and the reverse primer 5′- GGC AGA GGG TGC AGT GAG C-3′, for rs17878467 variant with forward primer 5′- TGG CAC CCT GTA AAG CTC TCC TG-3′ and reverse primer 5′- GGG CAA CGT CGG GGC AC -3′ [33], and for rs9904341 variant was performed using the forward primer F: 5′- CGT TCT TTG AAA GCA GTC GAG -3′ and the reverse primer 5′- TGT AGA GCG GTG GTC CT-3′ [22].

PCR for the rs8073069, rs17878467, and rs9904341 variants were performed for 35 cycles in a 10-μL volume containing 100 ng DNA, 10X buffer (500 mM KCl, 100 mM Tris- HCl, and 0.1% Triton TMX-100), 2.0 mM MgCl_2_, 200 μM dNTPs, 1 pM of each primer, and 2 U Taq DNA Polymerase. Denaturation was performed at 94 °C, the annealing temperature was at 63 °C for the three variants, and elongation was at 72 °C for 2 min each. Five microliters of the PCR product were digested with 4 U of *Eco0109* I, *Hae*II, and *Bst*UI restriction enzymes (New England Biolabs, Ipswich, MA, USA), respectively. The digested products were separated into 8% polyacrylamide gels. Fragments observed by electrophoresis corresponded to 164 bp for the polymorphic allele (C) and 145 bp and 19 bp for the wild-type allele (G) for the rs8073069 variant. For the rs9904341 variant, the fragments observed by electrophoresis corresponded to 329 bp for the polymorphic allele (C) and 234 bp and 92 bp for the wild-type allele (G). Finally, for rs17878467, the fragment observed by electrophoresis corresponded to 619 bp for the polymorphic allele (T) and 360 and 258 bp for the wild-type allele (C). Quality control for these assays was assessed in randomly selected samples re-genotyped by an independent technician. The concordance among genotype assays was 100%.

### 2.3. Statistical Analysis

Frequencies of alleles and genotypes were estimated through direct counting in both studied groups. The Hardy–Weinberg equilibrium (HWE) was assessed using the chi-square test. Differences in the distribution of alleles and genotypes, as well as the characteristics of the patient and control groups, were also examined by the chi-square test. To estimate the association between breast cancer and the presence of alleles or genotypes, we conducted stratified analyses examining the effects of age, smoking status, TNM stage, and histologic–molecular subtypes. A multivariate logistic regression analysis was performed. This analysis included the *BIRC5* variants along with confounding variables. The analysis was performed to ratify the association with breast cancer susceptibility. The odds ratio (OR) and its 95% confidence intervals (CIs) were calculated using SPSS v17.0 software (SPSS, Inc., Chicago, IL, USA). For all statistical analyses, statistical significance was defined as *p* < 0.05. A Bonferroni correction test was performed to adjust the *p*-values, considering *p* < 0.016 as the significant value. Power analyses confirmed that all tests had a minimum statistical power of 80%. 

Haplotype analysis was performed by first inferring the combinations of these SNPs of *BIRC5* using the software program SHEsis plus. Haplotypes were then constructed and their frequencies compared between breast cancer cases and controls.

### 2.4. In Silico Analysis of Variants Analyzed

To assess the impact of genetic variants, we conducted an in silico analysis utilizing the Combined Annotation Dependent Depletion (CADD) (v1.7) tool, which evaluates the potential deleteriousness of variants by analyzing their positions in the human genome (GRCh38). CADD provides raw and C-PHRED scores, which estimate the likelihood of a variant being deleterious. Higher C-PHRED scores indicate a higher probability that the variant is deleterious. CADD incorporates over 60 annotations to categorize variants, including MotifE, mirSVR, tOverlap, and MMSplice_exon. 

The PolymiRTS, SpliceAI, and RegulomeDB databases were also consulted for information on microRNAs (miRNAs), splice alternatives, DNA motifs, and binding sites to transcription factors, respectively. The PolymiRTS database was utilized to supplement the existing data and identify microRNAs (miRNAs) associated with the untranslated regions (UTRs) of the analyzed variants. Gene Expression Profiling Interactive Analysis (GEPIA) and Genotype-Tissue Expression (GTEx) databases were employed for gene expression analysis across diverse tissues and developmental stages.

**Figure 1:**
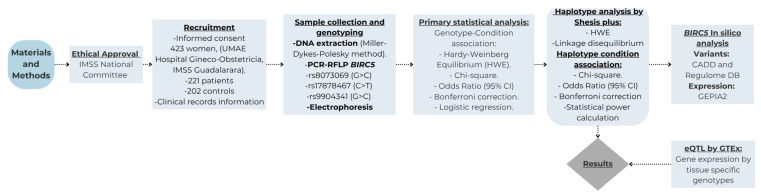
Flowchart summarizing the research design.

Additionally, an expression quantitative trait loci (eQTL) analysis was performed by correlating the genotypes of *BIRC5* gene polymorphisms with the gene expression levels obtained from GTEx. Robust regression models were applied to assess the association between variants and expression, which is natively implemented on the platform. Figure 1 Flowchart summarizing the research design.

## 3. Results

### 3.1. Characteristics of the Subjects Included in the Study 

The study included 423 women, of whom 221 had clinical and histological confirmation of breast cancer (BC); meanwhile, the control group consisted of 202 healthy female blood donors, all of whom were efficiently genotyped for the three variants analyzed. Table 1 compares the demographic data and clinicopathological characteristics of breast cancer (BC) patients and the control group. The mean ages were 55.93 and 57.41 years for the BC patients and the control group, respectively. Alcohol and tobacco consumption were not associated with BC (*p* > 0.05). In the BC group, 64.71% were in advanced stages III–IV, 36.65% were overweight, 42.54% had obesity, 94.57% showed unilateral tumors, 87.33% were of the histologic ductal type, 57.01% were molecular subtype luminal A, and 73.30% had positive metastatic node status. 

### 3.2. Genotype Frequencies and Haplotype Analysis of the BIRC5 Variants 

A comparative analysis of the *BIRC5* rs8073069 G>C, rs17878467 C>T, and rs9904341 G>C variants in breast cancer (BC) patients and the control group revealed significant differences (Table 2). For each variant analyzed, stratification analysis by age, smoking and alcohol consumption, TNM stage, histologic type, and histologic–molecular subtype was performed (Table 3, Table 4 and Table 5). In the control group, the analyzed variants were observed to be in Hardy–Weinberg equilibrium. For the rs8073069 variant, we observed that the patients who were carriers of C/C genotype showed an increased susceptibility for developing BC (OR = 2.87; 95% CI = 1.60–5.15, *p* = 0.001), and this association was also evident under the dominant model of inheritance (G/C+C/C vs. G/G) (OR = 2.08; 95% CI = 1.34–3.24, *p* = 0.001). Allelic frequencies were also significantly different, demonstrating that carriers of the C allele have increased susceptibility for developing BC (OR = 1.63; 95% CI = 1.24–2.14, *p* = 0.001) (Table 2). Regarding the association of this variant with clinical and pathological features, we observed that patients who were carriers of the G/C genotype under 50 years old showed an increased susceptibility to developing breast cancer (BC) (OR = 6.07; 95% CI = 1.57–23.39, *p* = 0.015) (Table 3). In the stratification analysis by TNM stage, histologic type, and histologic–molecular subtype, we observed that the patients with early TNM stage (TNM stage II) and carriers of G/C and C/C genotypes showed an increased susceptibility (OR= 3.34; 95% CI = 1.47–7.59, *p* = 0.004 and OR = 4.88; 95% CI = 1.90–12.5, *p* = 0.001), respectively. C/C genotype and advanced TNM stage (TNM IV) carriers exhibit increased susceptibility to BC (OR = 3.12; 95% CI = 1.38–7.03, *p* = 0.009). Concerning the histologic type, we observed that patients who were carriers of the C/C genotype showed an increased susceptibility with histologic ductal type (OR = 2.80; 95% CI = 1.54–5.08, *p* = 0.001) and the histologic–molecular subtypes, statistical significance was observed regarding the Luminal A subtype in the patients carrying the G/C and had an increased susceptibility to develop BC (OR = 2.31; 95% CI = 1.31–4.06, *p* = 0.004) and patients carries of C/C genotype and Luminal A and Her2 molecular subtype have an increased susceptibility to develop BC (OR = 2.92; 95% CI = 1.44–5.91, *p* = 0.004 and OR= 3.94; 95% CI = 1.41–11.0, *p* = 0.013) (Table 3). 

To understand the functional impact of the rs8073069 variant, in silico analysis using the Combined Annotation Dependent Depletion (CADD) tool has shown that the variant yielded a cumulative raw score of −0.57 and a C-PHRED score (a scaled metric derived from the raw CADD score; higher values indicate a higher predicted deleteriousness) of 0.11. Notably, the principal annotation assessed by CADD for this variant is the Conservation Score (ConsScore), which was 1.00, reflecting the conservation at this genomic position.

Regarding the rs17878467 variant, patients who were carriers of T/T genotypes showed increased susceptibility for developing BC (OR = 2.67; 95% CI = 1.42–5.00, *p* = 0.003), and this association was also evident under the dominant model of inheritance (C/T+T/T vs. C/C) (OR = 1.88; 95% CI = 1.24–2.85, *p* = 0.003). Allelic frequencies were also significantly different, demonstrating that carriers of the T allele have increased susceptibility for developing BC (OR = 1.56; 95% CI = 1.18–2.06, *p* = 0.001) (Table 2). In the stratification analysis by TNM stage, histologic type, and histologic–molecular subtype, we found that the patients with early (TNM II) and advanced TNM (TNM III) stages and carriers of C/T or T/T genotypes showed an increased susceptibility (OR = 2.49; 95% CI = 1.25–4.97, *p* = 0.012 and OR = 3.36; 95% CI = 1.44–7.829, *p* = 0.007), respectively. In the lobular histological type, T/T genotype carriers show an increased susceptibility to developing breast cancer (OR = 5.33; 95% CI = 1.53–18.50, *p* = 0.012). Non-statistically significant differences were observed in the histologic–molecular subtypes (Table 4).

**Figure 2:**
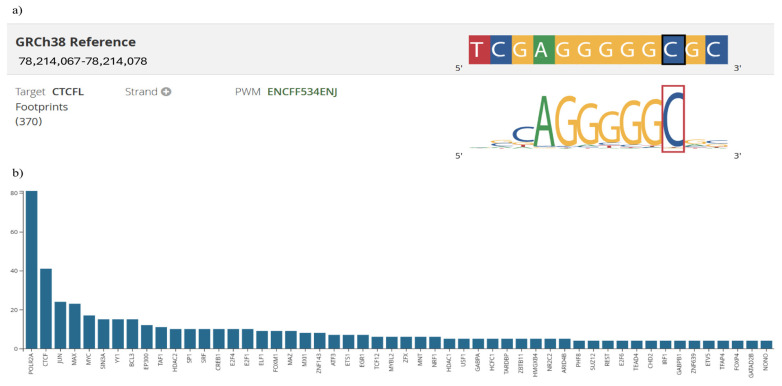
Regulatory Motif and Protein Binding Analysis of *BIRC5* Gene Variants. Panel (**a**): Identification of the CTCFL (BORIS) binding motif at the rs17878467 variant using RegulomeDB. The sequence highlighted indicates the position of rs17878467 within the motif predicted by CADD. Panel (**b**): ChIP-seq profile illustrating protein binding at the rs9904341 variant. The graph displays binding peaks, with POLR2A reaching up to 80 peaks, and notable binding by other transcription factors (*CTCF*, *JUN*, *MAX*, and *MYC*). Detailed statistical insights and interpretative commentary are provided in the Results section.

In silico analysis showed that this variant presented a raw score of 1.00 and a C-PHRED score of 10.35. The Conservation Score (ConsScore) was 1.00 among the annotations assessed by CADD. The motifE count was 1.00, reflecting that the variant affects a transcription factor binding motif. Additionally, the motifE HIpos was 0.00, indicating where the highest impact occurs in the affected motif. The motifE Score Change was −0.02, showing a change in the binding of the motif caused by the variant, which could alter the binding affinity of the transcription factor. The tOverlapMotif was 1.00, indicating if the variant overlaps with a known transcription factor binding motif. Lastly, the Motif Dist was 1.00, reflecting the variant location of the binding motif. Subsequently, using a RegulomeDB database revealed that the motif associated with this variant is a CTCFL (CCCTC-binding factor), formerly known as BORIS (Brother of Regulator of Imprinted Sites) (Figure 2a). This finding is corroborated by CADD prediction, which indicates the presence of a single regulatory motif in this region, suggesting a possible involvement in modulating *BIRC5* gene expression.

On the other hand, for the rs9904341 variant, we observed that patients who were carriers of the G/C and C/C genotype had an increased susceptibility for develop BC (OR = 2.52; 95% CI = 1.56–4.04, *p* = 0.001 and OR = 4.14; 95% CI = 2.27–7.54, *p* = 0.001), respectively; this association was also evident under the dominant model of inheritance (G/C+C/C vs. G/G) (OR = 2.87; 95% CI = 1.82–4.52, *p* = 0.001). Allelic frequencies were also significantly different, demonstrating that carriers of the C allele are more susceptible to developing BC (OR = 1.92; 95% CI = 1.46–2.53, *p* = 0.001) (Table 2). Regarding the rs9904341 variant with clinicopathological features, we observed that patients carrying the C/C genotype and with a history of alcohol consumption have an increased susceptibility to develop breast cancer (OR = 9.44; 95% CI = 1.92–46.35, *p* = 0.009). 

In the stratification analysis by the TNM stage, we found that the patients with TNM II and TNM III and carriers of C/C genotype showed an increased susceptibility to develop BC (OR = 4.49; 95% CI = 1.83–10.98, *p* = 0.001 and OR = 7.17; 95% CI = 2.72–18.91, *p* = 0.001). Concerning the histological type, carriers of the G/C genotypes and those with ductal histological type show an increased susceptibility to developing breast cancer (BC). On the other hand, statistical significance was observed for the Luminal A, Luminal B, and Her2 molecular subtypes in patients carrying the C/C genotype (Table 5). 

The analysis of the rs9904341 variant using the CADD tool yielded a raw score of 0.303 and a C-PHRED score of 3.31, accompanied by a Conservation Score of 3.00. The motifE Count was 4.00, suggesting that this variant may affect transcription factor binding motifs. The motifE HIpos value of 0.00 indicates where the highest impact occurs in the affected motifs, and the motifE Score Change of 0.01 suggests that the change in the binding score of the motifs is due to the variant. Furthermore, miRNA interaction was evaluated using the MirSVR CADD annotations, and the MirSVR score was −0.04, indicating a minimal predicted effect on microRNA-mediated regulation. The associated MirSVR-E energy score was −22.61, representing the free energy change due to microRNA binding, and the MirSVR-Aln alignment score was 143.00, reflecting the strength of the microRNA-target alignment. The CADD tool indicates that this variant modifies exon 1, and the MMSp_Exon value was 0.193, suggesting the maximum entropy-based splicing potential for the exon where the variant is located. These annotations indicate that rs9904341 may have a significant impact on splicing and regulatory mechanisms. In addition, the results were corroborated using Regulomedb, which revealed that the location in question is characterized by the presence of ChIP-seq information on regulatory proteins, accompanied by numerical values indicating each protein’s binding affinity and relevance. ChIP-seq analysis of the region corresponding to the rs9904341 variant revealed a distribution of 52 proteins, protein binding peaks ranging from 0 to 80. The signal of POLR2A is particularly noteworthy, with a maximum of 80 peaks, suggesting a high degree of transcriptional activity at this specific location. Furthermore, the presence of other factors, such as CTCF, JUN, MAX, and MYC (40, 20, 20, and 18, respectively), suggests a complex regulatory environment that may influence the differential expression of *BIRC5* (Figure 2b).

Regarding haplotype analysis, we identified eight distinct haplotypes in the *BIRC5* gene (Table 2). Our results indicate that rs8073069, rs17878467, and rs9904341 variants in the *BIRC5* gene are in linkage disequilibrium. Statistically significant differences were observed for one haplotype. The C allele combination in rs8073069, the T allele combination in rs17878467, and the C allele combination in rs9904341 may be risk factors for breast cancer (BC) (OR = 4.20; 95% CI = 2.38–7.41, *p* = 0.001). 

**Figure 3:**
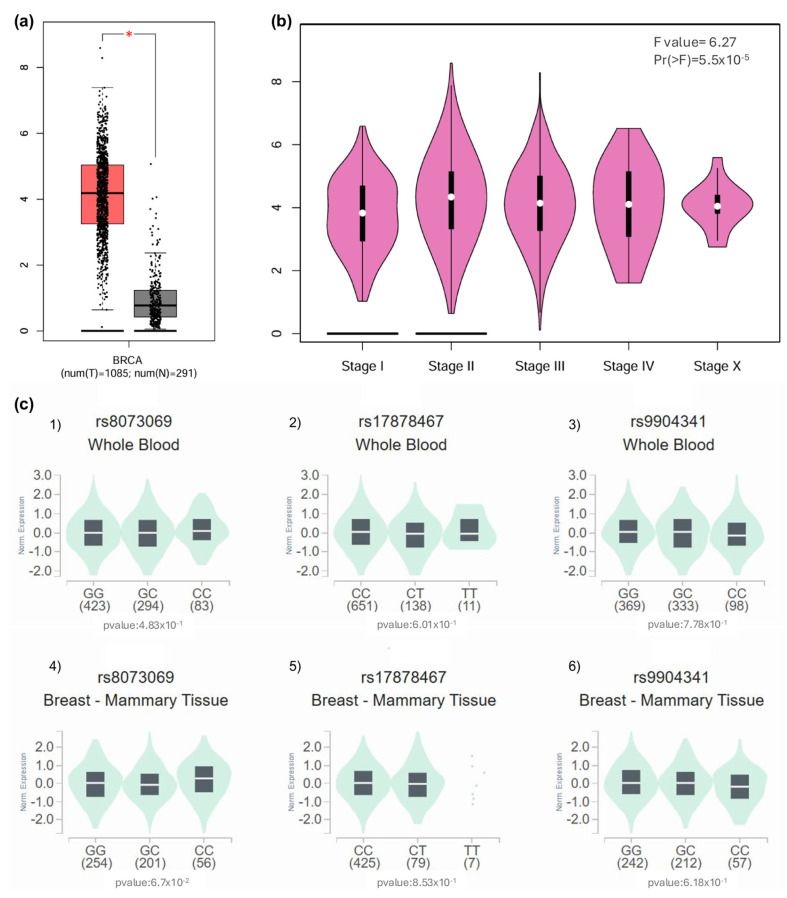
Analysis of *BIRC5* expression in breast cancer tissues and eQTL investigation of rs8073069, rs17878467, and rs9904341 variants. (**a**) Overexpression of *BIRC5* in Breast Cancer Tissue: The expression levels of *BIRC5* were examined in breast cancer (BRCA) tissue using the GEPIA (Gene Expression Profiling Interactive Analysis) database. The results indicate that *BIRC5* is significantly overexpressed in patients with breast cancer compared to normal breast tissue (*p*  <  0.01), suggesting a potential role of *BIRC5* in breast cancer pathogenesis. (**b**) *BIRC5* Expression Across Tumor Stages: An analysis of *BIRC5* expression across various tumor stages was conducted using the GEPIA database. The findings revealed significant variations in expression levels among the tumor stages (F value = 6.27, *p* < 0.001), indicating that *BIRC5* expression correlates with tumor progression and may serve as a marker for disease progression. (**c**) eQTL Analysis of *BIRC5* Variants in Whole Blood and Breast Tissue: (**c1**) Variant rs8073069: An eQTL (expression Quantitative Trait Loci) analysis was performed to assess the association between the rs8073069 variant in *BIRC5* and its expression levels. In whole blood samples, no significant association was observed (*p*  =  0.483). (**c2**) However, there was a trend toward significance (*p*  =  0.067) in breast tissue, suggesting a potential tissue-specific effect that warrants further investigation. (**c3**) Variant rs17878467: The same eQTL methodology was applied to the rs17878467 variant. The analysis showed no significant association with *BIRC5* expression in whole blood (*p*  =  0.601). (**c4**) The analysis showed no significant association with *BIRC5* expression in breast tissue (*p*  =  0.853), indicating that this variant may not significantly influence gene expression in these tissues. (**c5**) Variant rs9904341: The eQTL analysis for the rs9904341 variant revealed no significant association with *BIRC5* expression in whole blood (*p*  =  0.778). (**c6**) The analysis showed no significant association with *BIRC5* expression in breast tissue (*p*  =  0.618). These results suggest that rs9904341 may not have a substantial impact on the regulation of *BIRC5* expression in the examined tissues.

Regarding in silico expression analysis, *BIRC5* transcript levels were significantly elevated in breast carcinoma samples (TCGA-BRCA cohort) relative to normal mammary tissue (GTEx cohort; *p* < 0.01). The interquartile ranges, depicted in the boxplot, further underscored this disparity, with tumor medians exceeding typical tissue values, as illustrated in Figure 3a. In addition, staging analysis by GEPIA revealed a progressive increase in *BIRC5* expression from Stage I to IV tumors (ANOVA: F = 6.27, *p* < 0.001). The violin plots in Figure 3b capture this trend, showing a rightward shift in expression density peaks, particularly pronounced in stages III and IV. 

Genetic variant analysis yielded no significant eQTL associations for rs8073069, rs17878467, or rs9904341. In whole blood Figure 3(c1,c3,c5), genotype-stratified boxplots showed overlapping distributions across allelic variants (*p* > 0.4 for all comparisons). A similar outcome was observed in breast tissue, where none of the variants reached statistical significance, although rs8073069 approached marginal association (*p* = 0.067).

### 3.3. Multivariable Logistic Regression Analysis with Confounding Variables

Table 6 presents the results of the multiple logistic regression analysis, which includes confounding variables. In the presence of the three variants associated with *BIRC5* (rs8073069, rs17878467, and rs9904341), tobacco and alcohol consumption were not statistically significant, suggesting that these variables do not increase the risk or susceptibility to developing breast cancer (BC).

## 4. Discussion

The *BIRC5* gene is transcribed from a GC-rich promoter, lacking a TATA box, to produce an RNA transcript and a 142-amino-acid protein called survivin [5,34]. This protein, a member of the inhibitor of apoptosis proteins (IAPs) family, plays a crucial role in inhibiting the cell cycle (mitosis) and apoptosis. In the promoter region of the *BIRC5* gene many variants including rs8073069, rs17878467 and rs9904341 [5,28,31,35], have shown associations with different types of cancers, including leukemia [36,37], colorectal [38,39,40,41], renal [41], gastric [42,43,44], pancreatic [45], prostatic [17], urothelial [46], cervical [47], bladder [46], esophageal [48,49], nasopharyngeal [24], and breast cancer [50].

The present study investigated the association between genetic variants in the promoter region of the *BIRC5* gene and the risk of developing breast cancer in Mexican patients. The aim was to analyze the rs8073069, rs17878467, and rs9904341 variants of the *BIRC5* gene in patients with breast cancer to explore their potential as genetic risk markers in this population [2]. Notably, this is the first study in the Mexican population to investigate these specific variants.

The sociodemographic characteristics of the present study reveal notable trends in age at diagnosis. Specifically, the mean age at breast cancer diagnosis observed in the present study was 55.93. This finding contrasts with the global average age at breast cancer diagnosis, which is 63 years. These findings underscore the potential for regional disparities in breast cancer epidemiology and corroborate prior reports from the Mexican population, where the mean age at diagnosis is 52 years [51,52]. Concerning behavioral risk factors, the analysis revealed no significant association between alcohol or tobacco consumption and BC risk, which is consistent with the findings of Goldvaser et al. [53] who did not find an association between smoking and BC. In contrast, a multinational meta-analysis by Allahqoli et al. [54] identified smoking as a potential risk modifier in broader populations. The observed discrepancies may be attributable to variations in study design and genetic backgrounds. 

Our analysis of the *BIRC5* promoter variants suggests that the variant rs8073069 (G/C) contributes to an increased susceptibility to breast cancer, particularly in individuals carrying the C/C genotype and the C allele. These findings are consistent with previous research by Shi et al. [29], which reported lower survival rates in Swedish women. Conversely, Mashadiyeva et al. [31] and Sušac et al. [35] did not identify a significant association of this variant with breast cancer in Turkish and Croatian populations, respectively.

The location of this variant in the promoter region of *BIRC5* (rs8073069 G/C) reinforces its functional potential, as it has been linked to the expression of survivin, an apoptosis inhibitor implicated in tumor progression. This mechanism can be postulated to explain not only its association with breast cancer and its correlation with other types of cancers, such as non-small cell lung carcinoma (NSCLC), where *BIRC5* expression is associated with increased aggressiveness and poor prognosis [55].

The differential association of rs8073069 G>C with early stages suggests that *BIRC5* (Survivin) plays a key role in regulating apoptosis and cell proliferation. This could be explained by the overexpression of *BIRC5*, which plays a crucial role in evading apoptosis, potentially favoring the survival of early tumor cells. The role of *BIRC5* in apoptosis is to bind with caspases 3, 7, and 9, which are responsible for the initiation of apoptosis through cytochrome c, thereby leading to its suppression and interrupting the caspase cascade, and consequently decreasing apoptosis [56].

Notwithstanding the multiple studies that have demonstrated the relationship between *BIRC5* overexpression, especially in advanced stage III-IV TNMs, our results suggest that *BIRC5* is key in the early stages of mammary tumorigenesis and apoptosis evasion. This could be explained by the differences in molecular subtypes in our cohort, as there is evidence that *BIRC5* has a higher tendency to be expressed at early stages in Luminal B and HER2+ molecular subtypes. Adinew et al., 2022, similarly found a correlation of *BIRC5* overexpression in stage II breast cancer, a finding that is consistent with our results [57,58].

Key CADD annotations indicate that this variant is likely benign. A ConsScore of 1 suggests that the site is evolutionarily variable without compromising function, the raw score of –0.57 implies a low probability of deleterious effects, and a C-PHRED score of 0.11 supports minimal functional impact on the *BIRC5* gene.

Regarding the rs17878467 C/T in our study, we found that patients with the T/T genotype and those carrying the T allele of this variant had an increased susceptibility to developing breast cancer. This finding differs from that reported by Sušac et al. [35], who found no significant association between this variant and breast cancer in the Croatian population. Regarding other types of cancer, Yamak et al. [22] observed an increased risk of developing colon cancer in Turkey associated with this variant. The proposed mechanism to explain how the T allele contributes to cancer development involves an increase in promoter activity; the presence of the T allele could alter the balance between cell death and survival (by changing the apoptosis process), favoring the accumulation of malignant cells [22]. 

In silico analysis of the rs17878467 C/T variant indicates a low risk of deleterious effects. A raw score of 1.00 combined with a C-PHRED score of 10.35—despite placing the variant among the top 10% of predicted deleterious variants—suggests minimal impact on *BIRC5* function because its position is not highly conserved (Cons score = 1.00). Motif-related annotations show that the variant affects a single motif element (motifE count = 1.00) and overlaps with a known transcription factor binding site (tOverlap motif = 1.00). In addition, RegulomeDB identified the CTCFL (BORIS) motif associated with this variant. BORIS, a methylation-independent DNA-binding protein and paralog of CTCF, has been linked to increased tumor size and grade in several cancer types, potentially contributing to oncogenic transcription by counteracting the function [59].

On the other hand, the variant analyzed, rs9904341 G/C, in the present study has demonstrated that patients carrying the G/C and C/C genotypes, as well as those carrying the C allele, have an increased susceptibility to developing breast cancer. This finding is consistent with those reported by Motawi et al. [28], Aglan et al. [60], and Rasool et al. [5], who found a significant association between these genotypes and breast cancer in Egyptian and Indian populations, respectively. In contrast, the findings of Altiparmak et al. [30] and Mashadiyeva et al. [31] found no significant association of this variant with breast cancer in Turkish populations. Similarly, Rojhannezhad et al. [61] do not support this association, as they report no association of this variant in the Iranian population. These discrepancies could be attributed to genetic and environmental differences between populations, variations in sample size, and differences in study design.

The possible biological explanation for the association between the rs9904341 G>C variant and breast cancer susceptibility lies in its location at the cell cycle-dependent element (CDE) binding site and cell cycle homology regions (CHR) of the *BIRC5* gene promoter. This variant is associated with a modified binding affinity of the CDE/CHR repressor, resulting in altered promoter activity and, subsequently, differences in mRNA and survivin protein expression in both normal and cancer cells [23,24,25].

Specifically, the C allele has shown significantly elevated transcriptional activity compared to the G allele. Carriers of the C/C genotype have higher levels of *BIRC5* than those with the G/C and G/G genotypes [24,25,26]. Consequently, it is hypothesized that these functional variants in the promoter region of the *BIRC5* gene could contribute to individual susceptibility to develop tumors.

Supporting this theory, several epidemiological studies have demonstrated an association between the rs9904341 G>C variant and the risk of other types of cancer, such as lung [62], colorectal [63], urothelial [21], and gastric cancer [24]. Research has shown that this variant influences *BIRC5* expression, affecting key processes in cell proliferation and the inhibition of apoptosis [15].

Regarding the rs9904341, we observed that patients who were carriers of the C/C genotype and with a status of alcohol consumption had an increased susceptibility to develop BC. These results are consistent with the evidence that there is a strong association between alcohol consumption and the development of BC. Despite the amount of research that exists on this relationship, it has not been possible to clarify concretely what the specific mechanism or pathway of alcohol is that contributes to the development of breast cancer. Among the accepted and most researched hypotheses are the effect of alcohol consumption on increased levels of estrogen and receptors, release of acetaldehyde, and reactive oxygen species (ROS) [64].

Multiple international guidelines warn that alcohol consumption is a risk factor for developing BC, especially the Mexican clinical practice guideline, which is directly related to our population [65]. Some of the widely studied genes affected by alcohol are *BRCA1* and *BRCA2*, whose expression is attenuated, and HER2, whose expression is increased, as alcohol regulates specific genes of polymerase III [66,67].

In silico CADD analysis of the rs9904341 variant in *BIRC5* yielded a RawScore of 0.303 and a C-PHRED score of 3.31, indicating a low likelihood of deleterious effects, as scores below 10 are generally considered low risk. Motif-related annotations show that the variant affects four motif elements (motifE count = 4.00); however, a HIpos value of 0.00 suggests that critical binding positions remain unperturbed. Although RegulomeDB provided no specific annotations, highlighting current regulatory data gaps, ChIP-seq data confirm the presence of key regulatory proteins (e.g., POLR2A, CTCF, JUN, MAX, and MYC) at this locus. This observation suggests that the variant may subtly alter transcription factor binding and, consequently, affect *BIRC5* expression.

Furthermore, location-based CADD annotations indicate that rs9904341 may introduce a minor change in exon 1, as suggested by an MMSp_Exon score of 0.193, reflecting a slight potential impact on RNA splicing. Supporting this, Splice AI analysis detected no gain or loss of splice sites, while Pangolin analysis reported minimal probabilities for splice loss (0.01) and splice gain (0.02). Overall, these combined analyses suggest that rs9904341 is unlikely to impart significant deleterious effects or substantially alter RNA splicing, although its influence on gene regulation via transcription factor binding warrants further investigation.

Furthermore, haplotype analysis revealed that *BIRC5* variants (rs8073069, rs17878467, and rs9904341) were found to be in linkage disequilibrium, and two common haplotypes were identified, exhibiting statistically significant differences. Conversely, the combination of C-T-C alleles of rs8073069, rs17878467, and rs9904341 variants were associated with a high susceptibility to develop BC; on the other hand, G-T-G alleles of these variants were associated with a low susceptibility to establish BC, which contrasts with Mashadiyeva et al. (2023) [31], who conducted a haplotype analysis of rs8073069 and rs9904341 variants in the Turkish population determined no statistically significant differences between the haplotypes.

Finally, the differential expression of the *BIRC5* gene in breast cancer tissue was analyzed using the bioinformatics tool Gene Expression Profiling Interactive Analysis (GEPIA). The results showed that *BIRC5* is significantly overexpressed in breast cancer patients compared to normal breast tissue (*p* < 0.01) (Figure 3a). A subsequent analysis of *BIRC5* expression according to tumor stages revealed significant variation in its expression throughout cancer progression (F = 6.27, *p* < 0.001) (Figure 3b). This finding suggests that *BIRC5* plays a role not only in tumor initiation but also in disease progression, as indicated by the increase in its expression as the tumor stage advances. 

This finding is consistent with multiple studies that have identified *BIRC5*, also known as survivin, as a key factor in inhibiting apoptosis and promoting cell proliferation in various types of cancer, including breast cancer. The overexpression of *BIRC5* could contribute to tumor cell survival and resistance, thus favoring disease progression.

However, when the association between specific genetic variants of *BIRC5* and its gene expression was evaluated by eQTL analysis (quantitative expression of loci traits), it was observed that the variants rs8073069, rs17878467 and rs9904341 did not show a statistically significant association with *BIRC5* expression in either whole blood (*p* = 0.483 *p* = 0.601 and *p* = 0.778, respectively) or breast tissue (*p* = 0.067, *p* = 0.853 and *p* = 0.618, respectively) These results suggest that these variants may not directly affect the transcriptional regulation of *BIRC5* in the tissues analyzed.

The observed difference in *BIRC5* expression between cancerous and non-cancerous breast tissue may be attributed to the inclusion of non-tumor samples in the GTEx dataset, which can mask effects from the neoplastic microenvironment. GTEx’s reliance on a small number of samples and differences in cellular composition and normalization methods may also make it difficult to detect subtle changes. Finally, the functional impact of variants in the *BIRC5* promoter may only be seen under specific conditions [68].

Multivariate analysis with confounding variables showed that tobacco and alcohol consumption were not associated in patients with BC. In contrast to the international BC treatment guidelines, tobacco and alcohol consumption are considered to be one of the main risk factors [69,70].

## 5. Conclusions

In conclusion, our study demonstrates that the genotypes C/C of the rs8073069 variant, T/T of the rs17878467 variant, and G/C and C/C of the rs9904341 variant of the *BIRC5* gene are associated with an increased risk of breast cancer. Furthermore, some genotypes are also significantly associated with the TNM stage, the histology-molecular subtypes, and the age of diagnosis of BC patients. 

These results indicate promising potential for future research, which could involve integrating in vivo and in vitro mechanistic analyses. Such analyses might encompass promoter assays and the correlation between mRNA expression and genotype in tumor samples. Further investigation of these aspects will provide insight into the potential influence of these single-nucleotide polymorphisms (SNPs) on transcriptional regulation. In addition, these findings may contribute to a more comprehensive understanding of the genetic mechanisms underlying breast cancer pathogenesis and *BIRC5* susceptibility. 

A limitation of our study is the absence of follow-up data and treatment response outcomes. Additionally, evaluations—such as in vivo and in vitro analyses, promoter assays, or correlations between mRNA expression and genotype in tumor samples—were not conducted. These limitations highlight the need for future research to investigate whether these SNPs affect transcriptional regulation or interact with other molecular factors. Additional studies are necessary to confirm and extend these observations, further validating our findings given their potential value in predicting future outcomes [44,49,56].

## Figures and Tables

**Table 1:** Clinicopathological data of breast cancer patients and the control group.

Characteristics	Breast Cancer Group n = 221 (100%)	Control Group n = 202 (100%)	*p* Value
Age mean	55.93 (SD 8.90)	57.41 (SD 18.72)	0.850
Age			
<50	43 (19.46)	15 (7.43)	**0.001**
>50	178 (80.54)	187 (92.57)	
Alcohol			
Yes	44 (19.90)	27 (13.37)	0.072
No	177 (80.10)	175 (86.63)	
Tobacco			
Yes	42 (19.00)	27 (13.37)	0.117
No	179 (81.00)	175 (86.63)	
Body Mass Index (BMI) mean	29.28(SD 5.62)		
Normal	46 (20.81)		
Overweight	81 (36.65)		
Obesity	94 (42.54)		
Breastfeeding			
No	51 (23.08)		
Yes	170 (76.92)		
<6 months	36 (16.29)		
>6 months	134 (60.63)		
Hysterectomy			
Yes	46 (20.82)		
No	175 (79.19)		
TNM Stage			
I	13 (5.88)		
II	65 (29.41)		
III	64 (28.96)		
IV	79 (35.75)		
Tumor location			
Unilateral	209 (94.57)		
Left	113 (51.13)		
Right	96 (43.44)		
Bilateral	12 (5.43)		
Histology (adenocarcinoma)			
Ductal	193 (87.33)		
Lobular	25 (11.31)		
Mixed	3 (1.36)		
Molecular subtype			
Luminal A	126 (57.01)		
Luminal B	47 (21.27)		
Her2	31 (14.03)		
Triple-negative	17 (7.69)		
Metastatic node status			
Positive	162 (73.30)		
Negative	59 (26.70)		
Metastasis			
Yes	83 (37.56)		
No	138 (62.44)		

*p*-values were adjusted by the Bonferroni test (0.016); bold text highlights statistically significant findings.

**Table 2:** Distribution of genotypes, allelic frequencies, and haplotypes of the *BIRC5* rs8073069, rs17878467, and rs9904341 variants.

Genotype	BC Group n = 221 (100%)	Control Group n = 202 (100%)	OR (95% CI)	*p* Value
*BIRC5* rs8073069 G>C				
G/G	44 (19.91)	69 (34.16)	1.00 (Reference)	
G/C	122 (55.20)	103 (50.99)	1.85 (1.17–2.94)	0.011
C/C	55 (24.89)	30 (14.85)	**2.87 (1.60–5.15)**	**0.001**
G/C + C/C vs. G/G	177 (80.09)	133 (65.84)	**2.08 (1.34–3.24)**	**0.001**
Allele				
G	210 (47.50)	241 (59.70)	1.00 (Reference)	
C	232 (52.50)	163 (40.30)	**1.63 (1.24–2.14)**	**0.001**
*BIRC5* rs17878467 C>T				
C/C	57 (25.80)	80 (39.60)	1.00 (Reference)	
C/T	124 (56.11)	101 (50)	1.72 (1.12–2.64)	0.017
T/T	40 (18.09)	21 (10.40)	**2.67 (1.42–5.00)**	**0.003**
C/T + T/T vs. C/C	164 (74.20)	122 (60.40)	**1.88 (1.24–2.85)**	**0.003**
Allele				
C	238 (53.80)	261 (64.60)	1.00 (Reference)	
T	204 (46.20)	143 (35.40)	**1.56 (1.18–2.06)**	**0.001**
*BIRC5* rs9904341 G>C				
G/G	37 (16.74)	74 (36.63)	1.00 (Reference)	
G/C	126 (57.02)	100 (49.50)	**2.52 (1.56–4.04)**	**0.001**
C/C	58 (26.24)	28 (13.87)	**4.14 (2.27–7.54)**	**0.001**
G/C + C/C vs. G/G	184 (83.26)	128 (63.37)	**2.87 (1.82–4.52)**	**0.001**
Allele				
G	200 (45.20)	248 (61.40)	1.00 (Reference)	
C	242 (54.80)	156 (38.60)	**1.92 (1.46–2.53)**	**0.001**
Haplotype *BIRC5* rs8073069, rs17878467, rs9904341				
G-C-G	52.08 (0.118)	92.15 (0.228)	1.00 (Reference)	-
C-C-C	61.58 (0.139)	55.79 (0.138)	1.01 (0.68–1.49)	0.956
C-T-C	64.65 (0.146)	15.83 (0.039)	4.20 (2.38–7.41)	0.001
C-C-G	57.04 (0.129)	55.23 (0.137)	0.93 (0.62–1.39)	0.742
C-T-G	48.72 (0.110)	36.18 (0.090)	1.26 (0.80–1.98)	0.317
G-C-C	67.30 (0.152)	57.86 (0.143)	1.07 (0.73–1.57)	0.711
G-T-C	48.46 (0.110)	26.55 (0.066)	1.75 (1.06–2.86)	0.024
G-T-G	42.16 (0.095)	64.44 (0.160)	0.55 (0.36–0.84)	0.005

*p*-values were adjusted by the Bonferroni test (0.016); bold text highlights statistically significant findings.

**Table 3:** Association of the *BIRC5* rs8073069 G>C variant with demographic and clinical variables.

*BIRC5* rs8073069 G>C						
BC/Control				OR (95% CI); *p* Value		
Variable	GG	GC	CC	GC vs GG	CC vs GG	GC + CC vs GG
Age (years)						
<50	8/9	27/5	8/1	**6.07 (1.57–23.39); 0.015**	9.00 (0.91–88.5); 0.096	**6.56 (1.81–23.7); 0.006**
>50	36/60	95/98	47/29	1.61 (0.97–2.66); 0.078	**2.70 (1.45–5.02); 0.002**	**1.86 (1.15–3.00); 0.014**
Smoking status						
Yes	7/5	27/19	8/3	1.01 (0.27–3.68); 1.000	1.90 (0.32–11.0); 0.775	1.13 (0.32–4.02); 1.000
Drinking status						
Yes	10/5	24/18	10/4	0.66 (0.19–2.79); 0.734	1.25 (0.25–6.06); 1.000	0.77 (0.23–2.56); 0.902
TNM stage						
I + II	10/69	47/103	21/30	**3.14 (1.49–6.64); 0.003**	**4.83 (2.03–11.4); 0.001**	**3.52 (1.70**–**7.28); 0.001**
III + IV	34/69	75/103	34/30	1.47 (0.88–2.45); 0.165	**2.30 (1.21–4.36); 0.015**	1.66 (1.02–2.69); 0.050
I	2/69	7/103	4/30	2.34 (0.47–11.62); 0.470	4.60 (0.79–26.4); 0.161	2.85 (0.61–13.23);0.275
II	8/69	40/103	17/30	**3.34 (1.47–7.59); 0.004**	**4.88 (1.90–12.5); 0.001**	**3.69 (1.66**–**8.18); 0.001**
III	20/69	29/103	15/30	0.97 (0.50–1.85); 1.000	1.72 (0.77–3.81); 0.252	1.14 (0.62–2.08); 0.078
IV	14/69	46/103	19/30	2.20 (1.12–4.30); 0.029	**3.12 (1.38–7.03); 0.009**	**2.40 (1.26**–**4.59); 0.010**
Histologic type						
Ductal	41/69	102/103	50/30	1.66 (1.03–2.67); 0.045	**2.80 (1.54–5.08); 0.001**	**1.92 (1.22–3.01; 0.005**
Lobular	3/69	17/103	5/30	3.79 (1.07–13.44); 0.050	3.83 (0.86–17.0); 0.140	**3.80 (1.10–13.15);0.023**
Mixed	0/69	3/103	0/30	-----	-----	-----
Histologic–molecular subtype						
Luminal A	22/69	76/103	28/30	**2.31 (1.31–4.06); 0.004**	**2.92 (1.44–5.91); 0.004**	**2.45 (1.42–4.22); 0.001**
Luminal B	10/69	25/103	12/30	1.67 (0.75–3.70); 0.275	2.76 (1.07–7.08); 0.055	1.91 (0.90–4.09); 0.124
Her2	7/69	12/103	12/30	1.14 (0.43–3.06); 0.976	**3.94 (1.41–11.0); 0.013**	1.77 (0.72–4.33); 0.282
Triple Negative	5/69	9/103	3/30	1.20 (0.38–3.75); 0.968	1.38 (0.30–6.14); 0.979	1.24 (0.42–3.67); 0.896

*p*-values were adjusted by the Bonferroni test (0.016); bold text highlights statistically significant findings.

**Table 4:** Association of the *BIRC5* rs17878467 C>T variant with demographic and clinical variables.

*BIRC5* rs17878467 C>T						
	BC/Control			OR (95% CI); *p* Value		
Variable	CC	CT	TT	CT vs CC	TT vs CC	CT + TT vs CC
Age (years)						
<50	8/4	24/9	11/2	1.33 (0.32–5.53); 0.980	2.75 (0.40–18.87); 0.561	1.59 (0.40–6.31); 0.769
>50	49/76	100/92	29/19	1.68 (1.06–2.66); 0.033	2.36 (1.19–4.67); 0.019	**1.80 (1.16–2.79); 0.011**
Smoking status						
Yes	11/15	24/11	7/1	2.97 (1.03–8.55); 0.073	9.54 (1.02–89.22); 0.066	3.52 (1.26–9.81); 0.027
Drinking status						
Yes	10/15	23/10	11/2	3.45 (1.15–10.27); 0.046	8.25 (1.49–45.43); 0.022	**4.25 (1.50–11.97); 0.010**
TNM stage						
I + II	20/80	46/101	12/21	1.82 (0.99–3.32); 0.068	**2.28 (0.96–5.41); 0.090**	1.90 (1.06–3.40); 0.040
III + IV	37/80	78/101	28/21	1.66 (1.02–2.72); 0.052	**2.88 (1.45–5.73); 0.003**	**1.87 (1.17–3.00); 0.011**
I	7/80	5/101	1/21	0.56 (0.17–1.84); 0.513	0.54 (0.06–4.67); 0.916	0.56 (0.18–1.73); 0.469
II	13/80	41/101	11/21	**2.49 (1.25–4.97); 0.012**	3.22 (1.26–8.21); 0.023	**2.62 (1.34–5.12); 0.006**
III	17/80	32/101	15/21	1.49 (0.77–2.87); 0.302	**3.36 (1.44–7.82); 0.007**	1.81 (0.97–3.37); 0.081
IV	20/80	46/101	13/21	1.82 (0.99–3.32); 0.068	2.47 (1.06–5.77); 0.057	1.93 (1.08–3.45); 0.034
Histologic type						
Ductal	52/80	109/101	32/21	1.66 (1.06–2.58); 0.031	**2.34 (1.22–4.49); 0.015**	**1.77 (1.16–2.71); 0.010**
Lobular	5/80	13/101	7/21	2.05 (0.70–6.01); 0.274	**5.33 (1.53–18.50); 0.012**	2.62 (0.94–7.27); 0.090
Mixed	0/80	2/101	1/21	-----	-----	-----
Histologic–molecular subtype						
Luminal A	31/80	72/101	23/21	1.83 (1.10–3.07); 0.026	2.82 (1.37–5.82); 0.073	**2.00 (1.22–3.29); 0.007**
Luminal B	11/80	26/101	10/21	1.87 (0.87–4.01); 0.148	3.46 (1.29–9.24); 0.021	2.14 (1.03–4.46); 0.056
Her2	7/80	20/101	4/21	2.26 (0.91–5.61); 0.112	2.17 (0.58–8.14); 0.425	2.24 (0.92–5.46); 0.104
Triple Negative	8/80	6/101	3/21	0.59 (0.19–1.78); 0.509	1.42 (0.34–5.85); 0.912	0.73 (0.27–1.99); 0.730

*p*-values were adjusted by the Bonferroni test (0.016); bold text highlights statistically significant findings.

**Table 5:** Association of the *BIRC5* rs9904341 G>C variant with demographic and clinical variables.

*BIRC5* rs9904341 G>C						
	BC/Control			OR (95% CI); *p* Value		
Variable	GG	GC	CC	GC vs GG	CC vs GG	GC + CC vs GG
Age (years)						
<50	30/6	100/7	48/2	2.85 (0.89–9.15); 0.135	4.80 (0.90–25.34); 0.105	3.28 (1.08–9.93); 0.062
>50	7/68	26/93	10/26	2.71 (1.11–6.62); 0.039	**3.73 (1.28–10.85); 0.024**	**2.93 (1.24–6.96); 0.018**
Smoking status						
Yes	5/8	25/13	12/6	3.07 (0.83–11.32); 0.160	3.20 (0.72–14.14); 0.233	3.11 (0.89–10.84); 0.127
Drinking status						
Yes	6/10	21/14	17/3	2.50 (0.74–8.44); 0.233	**9.44 (1.92–46.35); 0.009**	3.72 (1.16–11.91); 0.045
TNM stage						
I + II	11/74	46/100	21/28	**3.09 (1.50–6.37); 0.002**	**5.04 (2.15–11.79); 0.001**	**3.52 (1.75–7.08); 0.001**
III + IV	26/74	80/100	37/28	**2.27 (1.33–3.88; 0.003**	**3.76 (1.93–7.30); 0.001**	**2.60 (1.55–4.34); 0.001**
I	1/74	8/100	4/28	5.92 (0.72–48.36); 0.128	10.57(1.13–98.71); 0.044	6.93 (0.88–54.43); 0.068
II	10/74	38/100	17/28	**2.81 (1.31–6.00); 0.006**	**4.49 (1.83–10.98); 0.001**	**3.17 (1.52–6.61); 0.002**
III	7/74	38/100	19/28	**4.01 (1.69–9.49); 0.001**	**7.17 (2.72–18.91); 0.001**	**4.70 (2.04–10.85); 0.001**
IV	19/74	42/100	18/28	1.63 (0.88–3.03); 0.157	2.50 (1.15–5.44); 0.032	1.82 (1.01–3.29); 0.060
Histologic type						
Ductal	30/74	110/100	53/28	**2.71 (1.64–4.48); 0.001**	**4.66 (2.50–8.71); 0.001**	**3.14 (1.93–5.09); 0.001**
Lobular	6/74	15/100	4/28	1.85 (0.68–4.99); 0.320	1.76 (0.46–6.71); 0.637	1.83 (0.70–4.78); 0.305
Mixed	1/74	1/100	1/28	0.74 (0.04–12.02); 1.000	2.64 (0.15–43); 1.000	1.15 (0.10–12.97); 1.000
Histologic–molecular subtype						
Luminal A	26/74	74/100	26/28	**2.10 (1.22–3.60); 0.009**	**2.64 (1.31–5.30); 0.009**	**2.22 (1.32–3.73); 0.003**
Luminal B	5/74	27/100	15/28	**3.99 (1.46–10.86); 0.007**	**7.92 (2.63–23.85); 0.001**	**4.85 (1.84–12.81); 0.001**
Her2	4/74	16/100	11/28	2.96 (0.95–9.21); 0.088	**7.26 (2.13–24.72); 0.001**	**3.90 (1.31–11.58); 0.003**
Triple Negative	2/74	9/100	6/28	3.33 (0.69–15.86); 0.202	**7.92 (1.51–41.63); 0.016**	4.33 (0.96–19.48); 0.071

*p*-values were adjusted by the Bonferroni test (0.016); bold text highlights statistically significant findings.

**Table 6:** Logistic regression analysis for the cases and controls in the *BC*.

Independent Variable	Regression Coefficient (B)	Standard Error	*p* Value (*p*)	OR (95% IC)
Smoking status Yes vs. No	0.402	0.283	0.156	1.49 (0.85–2.60)
Drinking status Yes vs. No	0.411	0.282	0.144	1.50 (0.86–2.62)
*BIRC5* rs8073069 GC + CC	**0.688**	**0.260**	**0.008**	**1.98** **(1.19–3.31)**
*BIRC5* rs17878467 CT + TT	**0.690**	**0.217**	**0.002**	**1.99** **(1.30–3.05)**
*BIRC5* rs9904341 GC + CC	**0.737**	**0.262**	**0.005**	**2.09** **(1.25–3.49)**
Constant	−2.142	0.457	**0.001**	
Model	X^2^ = 30.915, d.f. = 5, *p* = **0.001**			

*p*-values were adjusted by the Bonferroni test (0.016); bold text highlights statistically significant findings. X^2^ = Chi-square.

## Data Availability

The original contributions presented in this study are included in the article. Further inquiries can be directed to the corresponding author.

## Abbreviations

The following abbreviations are used in this manuscript:

BC	Breast cancer
PCR-RFLP	Polymerase chain reaction- restriction fragment length polymorphism
OR	Odds ratios
INEGI	Instituto Nacional de Estadística y Geografía
TNM	Tumor-node-metastasis
*BIRC5*	Baculoviral inhibitor of apoptosis repeat-containing 5
IAP	Inhibitor of apoptosis
CHR	Cell-cycle homology region
CDE	Cell-cycle dependent elements
miRNA	MicroRNAs
UMAE	Unidad Médica de Alta Especialidad
IMSS	Instituto Mexicano del Seguro Social
HWE	Hardy–Weinberg equilibrium
UTRs	Untranslated regions
Cis	Confidence intervals
CADD	Combined Annotation Dependent Depletion
GEPIA	Gene Expression Profiling Interactive Analysis
GTEx	Genotype-Tissue Expression
eQTL	Quantitative expression of loci traits
ConsScore	Conservation Score
CTCFL	CCCTC-binding factor
BORIS	Brother of Regulator of Imprinted Sites
NSCLC	Non-small cell lung carcinoma
ChIP-seq	Chromatin Immunoprecipitation Sequencing
AI	Artificial Intelligent
